# Stress Concentration Analysis of the Corroded Steel Plate Strengthened with Carbon Fiber Reinforced Polymer (CFRP) Plates

**DOI:** 10.3390/polym14183845

**Published:** 2022-09-14

**Authors:** Anbang Li, Hao Wang, Han Li, Deliang Kong, Shanhua Xu

**Affiliations:** 1 School of Civil Engineering, Xi’an University of Architecture & Technology, Xi’an 710055, China; 2 State Key Laboratory of Green Building in Western China, Xi’an 710055, China; 3 Key Laboratory of Structural Engineering and Earthquake Resistance, Ministry of Education, Xi’an 710055, China; 4 Central Research Institute of Building and Construction Co., Ltd., MCC Group, Beijing 100088, China

**Keywords:** corroded steel plate, carbon fiber-reinforced polymer, strengthening, stress concentration, finite element modeling

## Abstract

The purpose of this study is to investigate the stress concentration of a corroded steel plate strengthened with carbon fiber reinforced polymer (CFRP) plates. An accelerated corrosion experiment was first executed to acquire corroded steel plates, and then surface profile measurements were conducted to obtain 3D coordinate data of the corroded steel surface. Finite element models considering the surface morphology of the corroded steel plate and the interfacial bonding properties between the CFRP plate and the corroded steel plate were established to investigate the stress concentration of the corroded steel plate strengthened with and without CFRP plates. The reliability of the numerical modeling method was verified based on a mesh convergence analysis and a comparison of the fatigue test, 3D morphology scanning, and numerical analysis results. Specimens with five levels of corrosion damage, six kinds of CFRP-strengthening stiffness, five kinds of adhesive thickness, and five levels of CFRP prestress were numerically modeled. The primary indications consist of features of stress distribution, and the stress concentration factors *K*_t_ and *K*_tg_ were analyzed. Results showed that the features of stress distribution and the stress concentration factor *K*_t_ of the corroded steel plate strengthened with and without CFRP plates are only related to the shape, size, and position of rust pits, but not to the degree of uniform corrosion or the reinforcement parameters. The value of *K*_t_ for the corroded steel plate with a corrosion duration of 6~18 months and a weight loss rate of 9.16~21.78% was approximately 1.199~1.345. The converted stress concentration factor *K*_tg_ has more practical significance than the stress concentration factor *K*_t_ in describing the influence of corrosion and CFRP reinforcement on the peak tensile stress of the corroded steel plate. The value of *K*_tg_ increased linearly with the increase of the weight loss rate of the corroded steel plate and decreased appreciably with the increase of the strengthening stiffness and prestress level of the CFRP plates, and it presented a very small increasing trend with the increase of the adhesive thickness.

## 1. Introduction

Corrosion is almost an inevitable durability problem for steel structures in service, especially for those exposed long-term to an industrial, marine, or atmospheric corrosion environment [1]. Corrosion damage, which consists of uniform corrosion and pitting corrosion, not only causes an effective cross-section loss of the steel structure but also forms uneven rust pits on its surface, which will cause an increase in average stress and a significant stress concentration in the steel structure section, respectively. Several researchers have been primarily concerned with the effect of corrosion on the stress concentration of corroded steel structures. A stress concentration analysis of a pre-corroded Q690 high-strength steel plate was conducted by Jia et al. [2], and it was concluded that under the uniaxial tensile load, the corroded steel plate showed an obvious stress concentration effect, most nodes were in a multiaxial stress state, and both uniform and pitting corrosion induced a yield load and an ultimate load degradation. Xu et al. [3] conducted an experimental and numerical study to investigate the effects of corrosion pits on the fatigue life of pre-corroded Q235 steel plates and observed that a one-to-one correspondence exists between the stress and pit distribution, high stress is generally found in most of the valleys of the measured corroded steel surface, and plastic strains are found in the critical pit. Kodvanj et al. [4] carried out a numerical analysis of the stress concentration in non-uniformly corroded small-scale specimens of a transversely stiffened welded shipbuilding steel plate and showed that in most of the cases analyzed, one of the areas with increased stress identified from FE results coincides with the actual fatigue failure position obtained from experiments. The aforementioned studies have shown that the stress concentration caused by uneven rust pits of the corroded steel surface is the main reason for the reduction in plasticity, fracture toughness, and fatigue performance of corroded steel structures. In order to restore the bearing capacity or the fatigue performance of corroded steel structures, a feasible method would be to eliminate the effective cross-section loss and stress concentration caused by corrosion through various reinforcement technologies.

Carbon fiber-reinforced polymer (CFRP) materials, which possess the significant advantages of a high strength/weight ratio as well as excellent fatigue and corrosion resistance [5,6], have been widely studied and applied in the field of steel structure reinforcement. The effectiveness of externally bonding CFRPs to improve the static-bearing capacity of steel plates [7,8] and steel beams [9,10], the fatigue life of defected steel plates [11,12], steel beams [13,14], and welded joints [15,16], and the overall [17,18] and local stability performance [19,20] of steel columns has been verified by the existing research. A strengthening system with CFRP bonded to steel substrate has been proved to be more efficient, with a minimized additional permanent load, eliminated stress concentration, and higher durability than traditional repair and reinforcement methods such as welding, bolting, or riveting [21,22,23]. Recently, a few studies have been concerned with the applications of CFRP materials in the reinforcement of corroded steel structures. Chotickai [24], Li et al. [25,26], and Xu et al. [27] have investigated the bond properties between CFRP materials and pre-corroded steel plates. Sharaf et al. [28] conducted an experimental and numerical analysis of the behavior of corroded steel plates strengthened with thin-ply glass/carbon hybrid FRP composites. The combined flexural and bearing strength of rehabilitated corroded steel circular hollow sections (CHS) under quasi-static large deformation three-point bending and direct indentation using CFRP sheets have been tested by Elchalakani et al. [29,30]. Yousefi et al. [31] carried out an experimental and numerical study to investigate the failure mode and load-bearing capacity of corroded slender steel columns strengthened with CFRP under axial compressive loads. The effectiveness of externally bonding CFRP plates to extend the fatigue life of corroded steel plates has been verified by the authors’ recent study [32]. The fatigue life of the patched specimen that presented the most significant strengthening effectiveness showed an extension of more than 85.3 times with respect to the unpatched corroded steel plate and approximately two times more than the uncorroded steel plate, and it was found that the fatigue cracks in the corroded steel plates were strengthened with and without CFRP plates. Both were initiated at the bottom of the rust pit on the corroded steel surface. The above research has shown that the stress concentration on the rough surface of the steel structure caused by corrosion not only changes the bond behavior of the interface between CFRP materials and corroded steel substrate, but also affects the load-bearing capacity and fatigue performance of corroded steel structures strengthened with CFRP materials.

The classical metal fatigue fracture theory believes that the fatigue life of metal structures can be divided into the crack initiation life and the propagation life, whereby the crack initiation life mainly depends on the maximum stress amplitude of the thermal stress point [33]. As for corroded steel structures strengthened with CFRP materials, the crack initiation life mainly depends on the maximum stress amplitude of the corroded steel plate, which is determined by the stress concentration level at the rust pits. Obviously, both the corrosion degree of the steel plate and the CFRP strengthening configuration present a great impact on the stress concentration on the steel surface [32,34]. Therefore, a stress concentration analysis is the basis for studying the mechanism of improving the fatigue performance of corroded steel structures strengthened with CFRP materials, and it is also one of the fundamental issues of the fatigue-strengthening design of corroded steel structures. Wang et al. [35] and Wu et al. [36] analyzed the stress concentration of CFRP-strengthened open-hole steel plates, and the stress concentration factors were demonstrated to be reduced with an increase in the modulus of carbon fiber and the number of CFRP layers. However, as for the corroded steel plate, the initial damage caused by corrosion not only causes effective cross-section loss and uneven rust pits on its surface, but also changes the load transfer characteristics between CFRP materials and corroded substrate. Hence, the corroded steel plate strengthened with CFRP is bound to have a distinctive mechanism and distribution of stress concentration with respect to the CFRP-strengthened open-hole steel plate. At present, to the authors’ knowledge, due to the randomness of the distribution of rust pits on steel surfaces and the complexity of variation in the interfacial bonding properties between CFRP materials and corroded steel plates, the stress concentration of a corroded steel plate strengthened with CFRP materials has not been systematically investigated.

This study presents systemic research to investigate the stress concentration of the corroded steel plate strengthened with CFRP plates. An accelerated corrosion experiment was first executed to acquire corroded steel plates, and the outdoors exposure test methods for periodic water spray were adopted to simulate the corrosion process of steel structures in a general marine atmospheric environment. Surface profile measurements were then conducted to obtain 3D coordinate data of the corroded steel surface, and the topographic feature parameters of the corroded steel surface were calculated and analyzed. Moreover, the specimens’ dimension and configuration that was applied in the authors’ recent experimental study [32] was adopted as the prototype structure, and finite element models that consider the surface morphology of the corroded steel plate and the interfacial bonding properties between CFRP plates and corroded steel plates were established and verified to investigate the stress concentration of the corroded steel plates strengthened with CFRP plates. The effect of the corrosion duration, CFRP strengthening stiffness, adhesive thickness, and the prestress level of CFRP plates on the peak tensile stress distribution and stress concentration factors *K*_t_ and *K*_tg_ of the corroded steel plate strengthened with CFRP plates were analyzed. The outcomes of this study can provide meaningful references and essential data for the fatigue strengthening design of corroded steel structures strengthened with CFRP plates.

## 2. Experimental Procedure

### 2.1. Accelerated Corrosion Experiment

**Figure 1:**
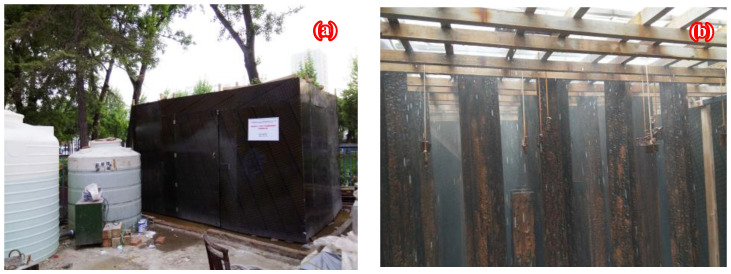
Walk-in natural environment accelerated corrosion test platform: (**a**) general view, (**b**) internal schematic diagram.

According to the Chinese codes “Corrosion of metals and alloys—Outdoors exposure test methods for periodic water spray” (GB/T 24517-2009) [37] and “Corrosion tests in artificial atmospheres—Salt spray tests” (GB/T 10125-2012) [38], the walk-in natural environment accelerated corrosion test platform was developed, as shown in Figure 1, and the outdoors exposure test methods for periodic water spray were adopted to simulate the corrosion process of steel structures in a general marine atmospheric environment. Hot-rolled H 350 × 175 × 7 × 11 beams with a strength grade of Q235 and a length of 2.0 m were stored in an accelerated test platform and sprayed with sodium chloride solution, of which the concentration and pH value were 50 mg/L and 6.2~7.2, respectively. Automatic sprinklers were set to spray every 4 h for 5 min, and five corrosion durations including 0, 6, 9, 15, and 18 months were preset for the hot-rolled H beams. After completing the preset corrosion process, fifteen corroded steel plates were cut from the flanges of the corroded H beams by wire-cut electrical discharge machining (WEDM) and adopted to conduct 3D surface profile measurements and a stress concentration analysis of the unenforced corroded steel plate and that strengthened with CFRP plates.

### 2.2. 3D Surface Profile Measurements

**Figure 2:**
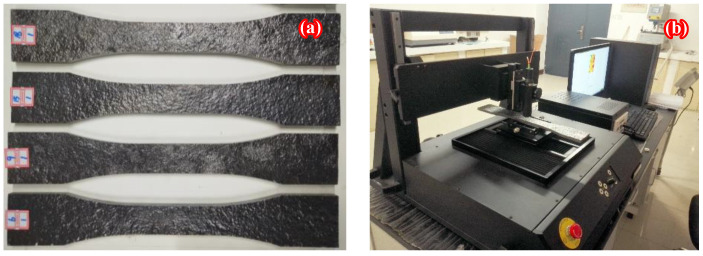

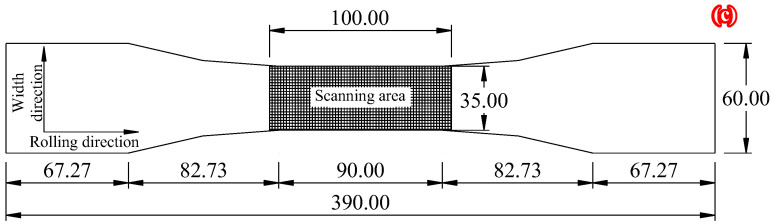
Three-dimensional surface profile measurements: (**a**) photograph of corroded steel plates after removing corrosion products, (**b**) photograph of surface morphology scanning test, (**c**) dimensions of cut dog-bone specimen and scanning area (mm).

Dog-bone-shaped corroded steel plates were cut from the flanges of corroded H beams by wire-cut electrical discharge machining (WEDM), the corrosion products were removed carefully by electric wire brushing, and the dust and greasy dirt on the steel surface were cleaned using anhydrous alcohol. Figure 2a illustrates the corroded steel plates after removing corrosion products. To provide morphology data of the rusted steel surface for the subsequent stress concentration analysis of the corroded steel plates strengthened with and without CFRP plates, a surface morphology scanning test of the corroded steel plates was first conducted using a non-contact 3D profiler ST-400 produced by NANOVEA (Irvine, CA, USA). The profiler, as shown in Figure 2b, consists of an optical measuring probe, a controller, an optical sensor, 3D data acquisition software, a data processing system, etc. The high-resolution surface topography measurements and real-time collection and processing of the 3D coordinate data of each measurement point were realized by white light co-aggregation technology. Figure 2c presents the dimensions of the cut dog-bone specimen and the scanning area. As illustrated in Figure 2c, the length and width of the parallel section for the dog-bone specimen were 90 mm and 35 mm, respectively, and the scanning area located in the middle of the specimen had dimensions of 100 mm × 35 mm, where 100 mm was formed by extending 5 mm to both sides based on the length of the parallel section of the specimen. The scanning step of the rolling direction and flange width direction were both 100 um; three-dimensional coordinate data of 1001 × 351 points can be collected for each corroded steel surface.

## 3. Experimental Results and Discussion

**Figure 3:**
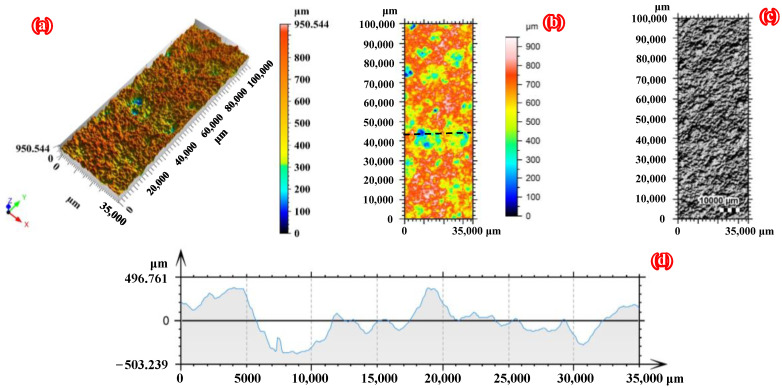
Three-dimensional morphology scanning results of the front side of C6-1: (**a**) 3D view of the surface, (**b**) pseudo color view of the surface, (**c**) photo copy, and (**d**) profile curve.

**Figure 4:**
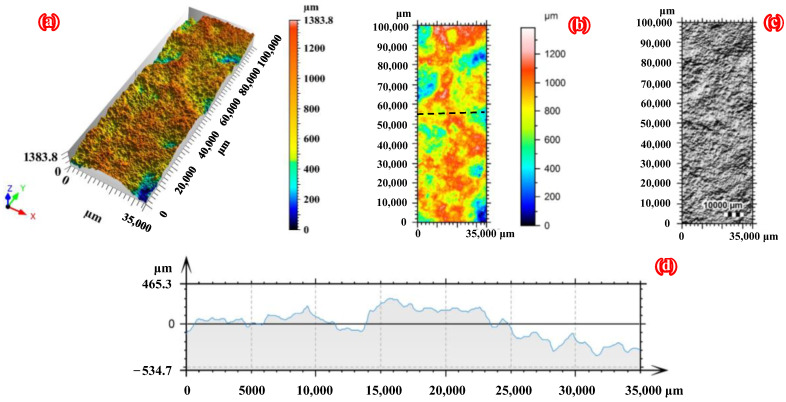
Three-dimensional morphology scanning results of the front side of C18-1: (**a**) 3D view of the surface, (**b**) pseudo color view of the surface, (**c**) photo copy, and (**d**) profile curve.

Figure 3 and Figure 4 clearly present the three-dimensional morphology scanning results of the corroded steel plates with a corrosion duration of 6 months and 18 months, respectively. As shown in Figure 3 and Figure 4, the surface corrosion morphology of corroded steel plates can be accurately restored using three-dimensional surface profile measurement technology. Moreover, the corroded steel substrates are covered with rust pits of different sizes due to uneven corrosion, which will inevitably lead to stress concentration in the steel plate. To quantitatively characterize the roughness of the corroded steel plates, the 3D roughness parameters, including the arithmetic mean height (*S*_a_), the root mean square height (*S*_q_), and the maximum height (*S*_z_), are selected and calculated by applying the following equations [39]:

(1) $$S_{a}=\frac{1}{A}\iint_{A}\left|z\left(x,y\right)\right|dxdy\approx \frac{1}{MN}\sum_{j=1}^{M}\sum_{i=1}^{N}\left|z\left(x_{i},y_{j}\right)\right|$$

(2) $$S_{q}=\sqrt{\frac{1}{A}\iint_{A}z^{2}\left(x,y\right)dxdy}\approx \sqrt{\frac{1}{MN}\sum_{j=1}^{N}\sum_{i=1}^{M}z^{2}(x_{i},y_{i})}$$

(3) $$S_{z}=\underset{(x,y)\in A}{\max}\left[z(x,y)\right]-\underset{(x,y)\in A}{\min}\left[z(x,y)\right]$$

where *A* is the nominal area of the scanning surface, *M* and *N* are the number of scan points in the rolling direction and width direction, respectively, and *z* (*x*, *y*) is the measured height of the scale-limited surface at position (*x*, *y*). Table 1 illustrates the results of the morphology calculation of the corroded samples, where *ξ* is the weight loss rate that can be deduced from the following equation:

(4) $$\xi =\left(W_{0}-W_{C}\right)/W_{0}\times 100\%$$

where $W_{C}$ and $W_{0}$ are the weight of the steel plates with and without corrosion damage.

**Figure 5:**
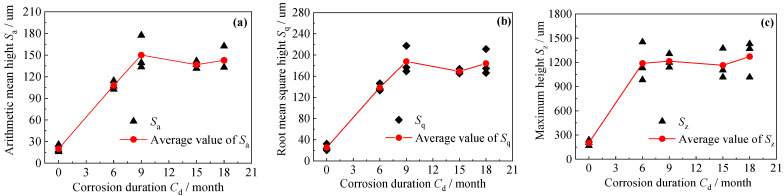
Effect of corrosion duration on the 3D roughness parameters: (**a**) arithmetic mean height *S*_a_, (**b**) root mean square height *S*_q_, and (**c**) maximum height *S*_z_.

Figure 5a–c illustrates the effect of the corrosion duration on the arithmetic mean height (*S*_a_), the root mean square height (*S*_q_), and the maximum height (*S*_z_) of the corroded steel surfaces, respectively. As shown in Figure 5, *S*_a_, and *S*_q_ showed a similar fluctuating variation process, that is, they both tended to increase at first with a rapid growth trend, then decrease, and at last increase again with the corrosion duration increased from 0 to 18 months. In addition, the arithmetic mean height (*S*_a_) and the root mean square height (*S*_q_) of the corroded steel plates were much greater than that of the uncorroded steel plates. The maximum height (*S*_z_), which reflected the two extreme distributions of the surface roughness, increased at first and then tended toward a steady value with the increasing corrosion duration. Our explanation for the above phenomenon may be summarized as follows: In the early stage of artificial accelerated corrosion (e.g., from 0 months to 6 months), with the anodic reaction due to the deposition and adsorption of chloride ions and forming localized pitting, the surface roughness of the corroded steel plates increased significantly compared with that of the uncorroded ones. With the further increase in corrosion duration (e.g., from 6 months to 9 months), the number of pitting pits increased, and then the rust layer appeared, the loose corrosion products turned dense and hard, and the function of the rust layer on the transmission of the corrosive medium may be changed from the initial growth to a definite retardation, and hence the roughness parameters *S*_a_, *S*_q_ and *S*_z_ increased with a relative gradual and slow growth trend. With the corrosion duration increasing from 9 months to 15 months, the chlorine and dissolved oxygen could hardly reach the bottom of the rust pits due to the increasing retardation of the rust layer, and thus the pitting pits grew mainly in the width direction. Therefore, the roughness parameters *S*_a_, *S*_q_, and *S*_z_ decreased somewhat. With the further increase in corrosion duration (e.g., from 15 months to 18 months), the corrosion pits grew alternately in the depth and width direction, and local pitting corrosion and uniform corrosion alternately played the dominant role on the surface topography and roughness of the steel substrates, resulting in the fluctuating variation process of *S*_a_, *S*_q_, and *S*_z_.

## 4. Finite Element Models of Corroded Steel Plate Strengthened with and without CFRP Plates

### 4.1. Mesh Generation

The key points of carrying out the stress concentration analysis of the corroded steel plates strengthened with CFRP plates are the reproduction of the surface morphology characteristics of corroded steel plates and the simulation of the interfacial bonding properties between CFRP plates and corroded steel plates in the numerical model. In this study, finite element software (ANSYS^®^14.5) (ANSYS, Inc., Pittsburgh, PA, USA) was employed to conduct the numerical study, and the finite element model (FEM) of “double layered variable-thickness shellspring-equal-thickness shell” was established to investigate the stress and stress concentration factor (SCF) of the corroded steel plate strengthened with and without CFRP plates.

**Figure 6:**
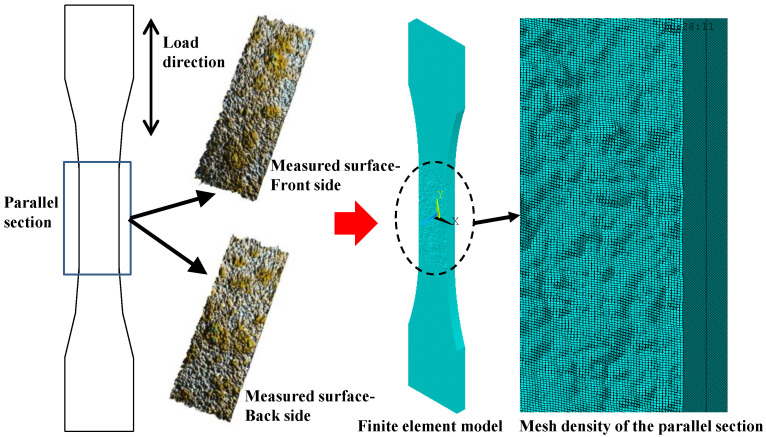
Reproduction process of the surface morphology characteristics of corroded steel plates in the finite element models.

Figure 6 depicts the reproduction process of the surface morphology characteristics of corroded steel plates in the finite element models. As shown in Figure 6, the rusted steel plate is simulated by adopting double-layered variable-thickness shell element SHELL91, that is, the corroded steel plate is composed of two layers of variable-thickness shells that correspond to the front and back side of the measured surfaces, respectively. SHELL91 is an eight-node nonlinear structural shell element, of which each node has six degrees of freedom, i.e., the translational displacement freedom along the x, y, and z directions of the node coordinate system and the rotational displacement freedom around each axis. The node offset control of the upper and lower shell elements is carried out by applying the element characteristic parameters KEYOPT (11), that is, the node of the upper shell element is offset to the bottom surface of the element, whereas the node of the lower shell element is offset to the top surface of the element. Reproduction of the surface morphology characteristics of the corroded steel plates is realized by importing surface corrosion depth data obtained by morphology scanning and assigning them to the real constant of element SHELL 91.

**Figure 7:**
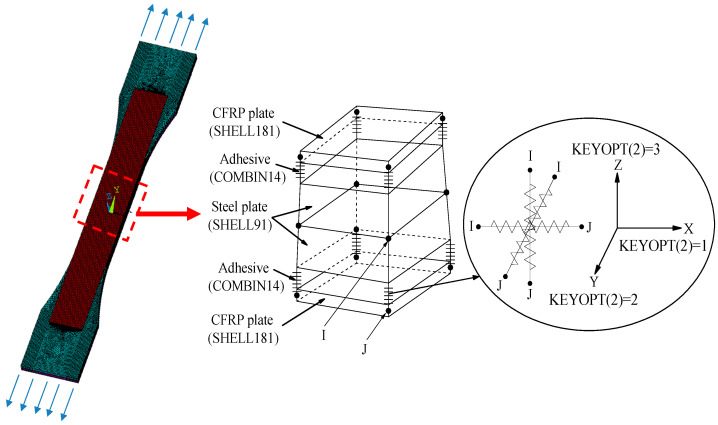
Element type and constraint relationship in the finite element models.

Figure 7 presents the element type and constraint relationship in the finite element models. As shown in Figure 7, the CFRP plate is simulated by the four-node shell element SHELL181 with equal thickness, and the adhesive layer between the CFRP plate and the corroded steel surface is simulated by the linear spring element COMBIN14, that is, three mutually perpendicular elements of COMBIN14 are inserted between the element nodes of the corroded steel plate and the CFRP plate, and the degree of freedom constraints in the x, y, and z directions is achieved by rewriting the element characteristic parameters KEYPOT (2).

### 4.2. Material Properties

**Figure 8:**
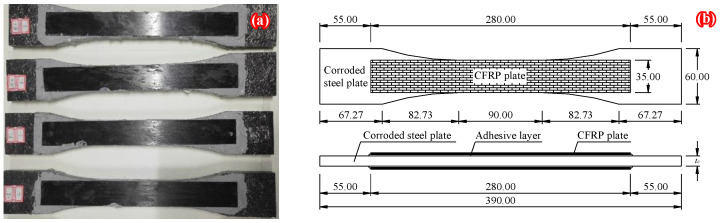
Prototype structures of the FEMs: (**a**) photograph of the specimens, (**b**) dimension and configuration of the corroded steel plate strengthened with CFRP plates.

Figure 8 illustrates the prototype structures of the finite element models (FEMs). As shown in Figure 8, the corroded steel plate double-side patched with CFRP plates, which was fatigue tested in the authors’ recent study [32], is adopted as the prototype structure to conduct a finite element analysis of the stress concentration. Considering that stress concentration is an elastic concept, the materials modeled in the FEMs are assumed to be linear elastic. The Modulus of elasticity and Poisson’s ratio of the steel plate were 181.9 GPa and 0.3, respectively. The thickness of the steel plate cut from the flange of the uncorroded H beams was 10.75 mm, and the Modulus of elasticity, tensile strength, and Poisson’s ratio of the CFRP plate were 165 GPa, 2400 MPa, and 0.3, respectively. The Modulus of elasticity and Poisson’s ratio of the adhesive layer were 5.3 GPa and 0.21, respectively.

The stiffness of the spring element, which reflects the bonding performance of the adhesive layer between the CFRP plate and the corroded steel plate, is defined by the real constant R (1) of the linear spring element COMBIN14, and the value of the spring stiffness can be deduced by a stress analysis of the adhesive layer [40]:

(5) $$\tau_{\mathrm{xz}}=\frac{\left|F_{x}\right|}{A}=\frac{G_{a}\left|u_{x}^{i}-u_{x}^{j}\right|}{t_{a}}$$

(6) $$\tau_{\mathrm{yz}}=\frac{\left|F_{y}\right|}{A}=\frac{G_{a}\left|u_{y}^{i}-u_{y}^{j}\right|}{t_{a}}$$

(7) $$\sigma_{\mathrm{zz}}=\frac{\left|F_{z}\right|}{A}=\frac{2\left(1-v_{a}\right)G_{a}\left|u_{z}^{i}-u_{z}^{j}\right|}{\left(1-2v_{a}\right)t_{a}}$$

where $\tau_{\mathrm{xz}}$ and $\tau_{\mathrm{yz}}$ are the shear stress of the adhesive layer in the X-Z plane and Y-Z plane, respectively; $\sigma_{\mathrm{zz}}$ is the tensile stress of the adhesive layer in the Z direction calculated according to the uniaxial strain assumption; $F_{x}$ and $F_{y}$ are the force of spring in the X and Y directions, respectively; $u_{x}^{i}$, $u_{y}^{i}$, and $u_{z}^{i}$ are the node displacements in the X, Y, and Z directions of node *i* coupled with the CFRP plate element node, respectively; $u_{x}^{j}$, $u_{y}^{j}$, and $u_{z}^{j}$ are the node displacements in the X, Y, and Z directions of node *j* coupled with the corroded steel plate element node, respectively; *A* is the area of adhesive represented by the spring element. $G_{a}$ is the shear modulus of the adhesive layer, $G_{a}=E_{a}/2(1+v_{a})$, where $E_{a}$ is the elastic modulus of the adhesive layer (5.3 GPa), $v_{a}$ is Poisson’s ratio of the adhesive layer (0.21), and $t_{a}$ is the actual thickness of the adhesive at the corresponding position of spring *i*-*j*.

According to Equations (5)–(7), the expressions of spring element stiffness in the X, Y, and Z directions can be obtained as follows:

(8) $$K_{x}=\frac{\left|F_{x}\right|}{\left|u_{x}^{i}-u_{x}^{j}\right|}=\frac{G_{a}A}{t_{a}}$$

(9) $$K_{y}=\frac{\left|F_{y}\right|}{\left|u_{y}^{i}-u_{y}^{j}\right|}=\frac{G_{a}A}{t_{a}}$$

(10) $$K_{z}=\frac{\left|F_{z}\right|}{\left|u_{z}^{i}-u_{z}^{j}\right|}=\frac{2\left(1-v_{a}\right)G_{a}A}{\left(1-2v_{a}\right)t_{a}}$$

**Figure 9:**
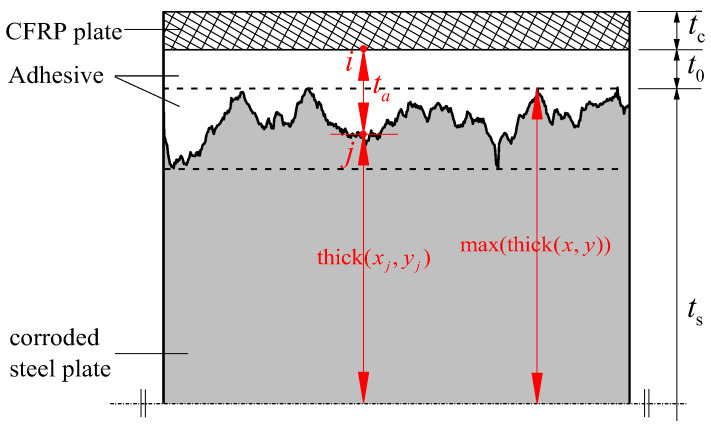
Diagrammatic sketch of the actual adhesive thickness corresponding to different positions on the surface of corroded steel plate.

Figure 9 presents a diagrammatic sketch of the actual adhesive thickness corresponding to different positions on the surface of the corroded steel plate. Considering the uneven morphology of the steel plate surface caused by corrosion, the actual thickness of the corresponding adhesive layer of each spring element, which mainly depends on the intended adhesive thickness and corrosion depth, is different, and the actual thickness of the adhesive at the corresponding position of spring *i*-*j* can be obtained using the following equation:

(11) $$t_{a,i-j}=t_{0}+\max(\mathrm{thick}(x,y))-\mathrm{thick}(x_{j},y_{j})$$

where $t_{0}$ is the intended adhesive thickness, max(thick(x,y)) is the maximum height of the entire corroded surface, and $\mathrm{thick}(x_{j},y_{j})$ is the scanning height of the corroded steel plate surface at the corresponding position of node *j*. Consequently, the influence of the uneven adhesive layer thickness that formed on the rough surface of the corroded steel plate on the interface bonding stiffness can be simulated by importing the surface corrosion depth data obtained by morphology scanning and assigning its influence on the spring stiffness to the real constant R (1) of element COMBIN14.

### 4.3. Boundary Condition and Load Application

The corroded steel plate strengthened with and without CFRP plates is subjected to a uniaxial tensile load at both ends. Unless otherwise specified, the tensile load applied to the models is 52.8 kN, which corresponds to the average value of the fatigue load adopted in the authors’ recent study [32]. The prestress of the CFRP plate, which is one of the main factors to be considered in the stress concentration analysis, is achieved by applying the temperature load to the CFRP plate, and the temperature load can be calculated by the following equation:

(12) $$\Delta T=\alpha f_{u}/\left(E_{c}\delta_{c}\right)$$

where α is the prestress level of the CFRP plate, which is defined as the ratio of the preset tensile stress to the tensile strength of the CFRP plate; $f_{u}$ and $E_{c}$ are the ultimate tensile strength and Modulus of elasticity of the CFRP plate, respectively; $\delta_{c}$ is the coefficient of linear expansion of the temperature of the CFRP plate, $\delta_{c}$ = 1 × 10^−5^/°C.

### 4.4. Model Validation and Mesh Convergence Analysis

**Figure 10:**
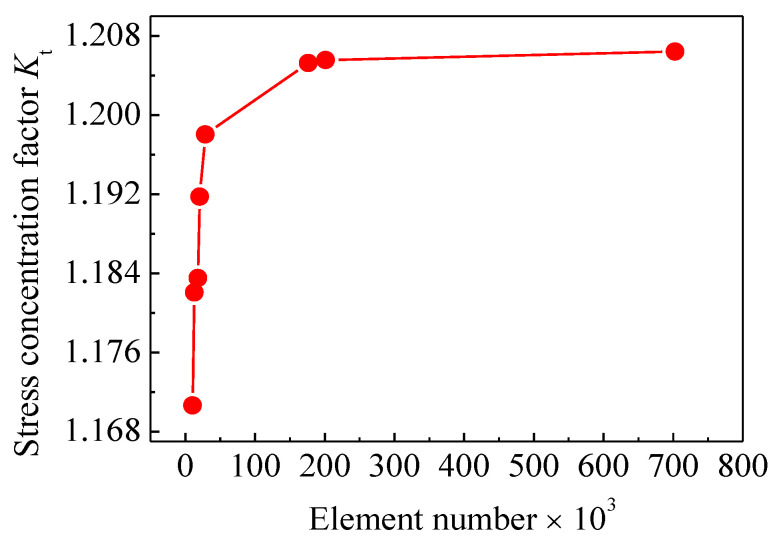
Variation of stress concentration factor with respect to element number.

To assure the mesh model is accurate enough, a mesh convergence study was first carried out to ensure that the SCF in the pit region of the corroded steel plate was convergent before the series of stress concentration analyses. Several element numbers for the mesh size of the FEMs were performed for the corroded steel plate of C6-1, and the variation in SCF with respect to element number is presented in Figure 10. As shown in Figure 10, the SCF response is almost convergent for a mesh number of 200,000. In this study, the simulation models for the corroded steel plate strengthened with and without CFRP plates were constructed with nearly 341,000 and 260,000 elements, respectively.

**Figure 11:**
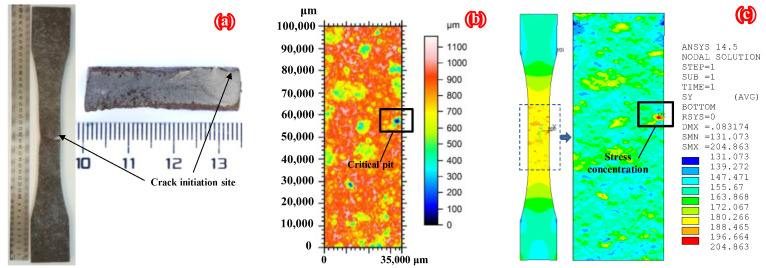
Comparison of (**a**) fatigue test, (**b**) 3D morphology scanning, and (**c**) FEM results of specimen C6-U-S1.

**Figure 12:**
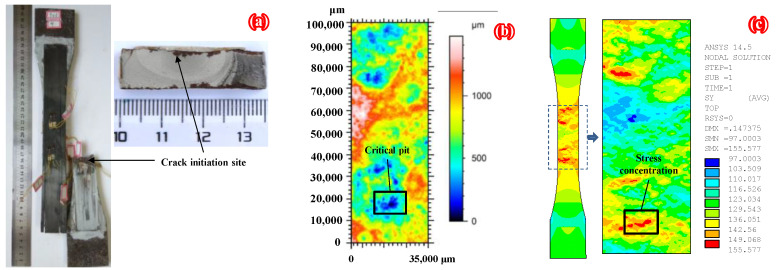
Comparison of (**a**) fatigue test, (**b**) 3D morphology scanning, and (**c**) FEM results of specimen C9-DS1-A.

To further verify the reliability of the numerical modeling method based on the 3D topography data proposed in this paper, the fatigue test results of specimens C6-U-S1 and C9-DS1-A in the authors’ recent study [32] were adopted for comparison with the three-dimensional morphology scanning and FEM results in this paper (see Figure 11 and Figure 12, respectively, where specimen C6-U-S1 is a bare corroded steel plate without any patch, and specimen C9-DS1-A is a corroded steel plate double-side patched with CFRP plates with a thickness of 1.4 mm). As shown in Figure 11 and Figure 12, the fatigue crack initiation site on the corroded steel plates strengthened with and without CFRP plates and the critical pit (i.e., the rust pit corresponding to the maximum pit depth) on the 3D surface topography present an excellent correlation with the stress concentration site in FEMs. This indicates that the FEMs applied in the present study can reproduce the surface morphology of the corroded steel plate and predict the location of stress concentration with reasonable accuracy.

## 5. Parameter Analysis

To further investigate the influence of corrosion and CFRP reinforcement on the stress concentration of corroded steel plates strengthened with and without CFRP plates, forty-four finite element models were established to carry out a parameter analysis of SCFs, and five levels of corrosion damage for the steel plates, six kinds of CFRP strengthening stiffnesses, five kinds of adhesive thicknesses, and five levels of CFRP prestress are considered in this section. A summary of the specimens’ parameters is presented in Table 2; the naming rules of the specimens are as follows: taking specimen C15-DS1.0-A0.5-PS4.5 as an example, C15 is a corrosion duration of 15 months, DS1.0 is double-side patched with CFRP plates with a thickness of 1.0 mm, A0.5 is the adhesive thickness (0.5 mm), and PS4.5 is the CFRP prestress level (4.5% of the tensile strength of the CFRP plate). *R*_s_ is the strengthening stiffness ratio of the patched specimen, which can be deduced from the following equation:

(13) $$R_{s}=E_{c}/E_{s}$$

where $E_{c}$ and $E_{s}$ are the stiffness of the external patched CFRP plates and corroded steel plate, respectively. *α* is the prestress level of the CFRP plate, which can be deduced from the following equation:

(14) $$\alpha =f_{\mathrm{pr}}/f_{u}$$

where $f_{\mathrm{pr}}$ and $f_{u}$ are the preset tensile stress and the ultimate tensile strength of the CFRP plate, respectively.

The results summary of the stress concentration analysis of the corroded steel plates strengthened with and without CFRP plates is presented in Table 1 and Table 2, respectively. The theoretical SCF *K*_t_ is defined as the ratio of the peak stress $\sigma_{\mathrm{peak}}$ at the rust pit on the surface of corroded steel plate to the nominal stress $\sigma_{\mathrm{nominal}}$ of the net section of the corroded steel plate that corresponds to the position of peak stress:

(15) $$K_{t}=\frac{\sigma_{\mathrm{peak}}}{\sigma_{\mathrm{nominal}}}$$

where $\sigma_{\mathrm{nominal}}$ can be deduced from the following equation:

(16) $$\sigma_{\mathrm{nominal}}={P/A}_{\mathrm{normal}}$$

where *P* is the applied tensile load, $A_{\mathrm{nominal}}$ is the area of the net section of the corroded steel plate that corresponds to the position of peak stress, and $A_{\mathrm{nominal}}$ can be calculated by the integration of the 3D topography scanning data on the cross section corresponding to the position of the peak stress.

*K*_t_ is essentially an elastic concept that gives a direct indication of the severity of the stress concentration and represents an amplification factor on the stress level. Furthermore, the converted SCF *K*_tg_, which is defined as the ratio of the peak stress $\sigma_{\mathrm{peak}}$ at the rust pit on the surface of the corroded steel plate to the gross stress $\sigma_{0}$ of the uncorroded, unstrengthened steel plate, is also informative to understand the influence of corrosion (negative part) and CFRP plate reinforcement (positive part) on the peak stress of the corroded steel plate strengthened with CFRP plates:

(17) $$K_{\mathrm{tg}}=\frac{\sigma_{\mathrm{peak}}}{\sigma_{0}}$$

where $\sigma_{0}$ can be deduced from the following equation:

(18) $$\sigma_{0}={P/A}_{0}$$

where $A_{0}$ is the cross-sectional area of the uncorroded steel plate. It is obvious that the two aforementioned stress concentration factors are interrelated. As for the unpatched corroded steel plate, $K_{\mathrm{tg}}$ > $K_{t}$ due to $\sigma_{\mathrm{nominal}}$ > $\sigma_{0}$, whereas for the corroded steel plate patched with CFRP plates, the size relationship between $K_{t}$ and $K_{\mathrm{tg}}$ becomes uncertain.

### 5.1. Effect of Corrosion Duration

**Figure 13:**
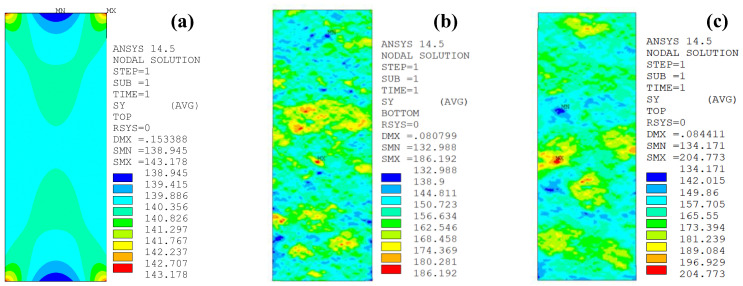

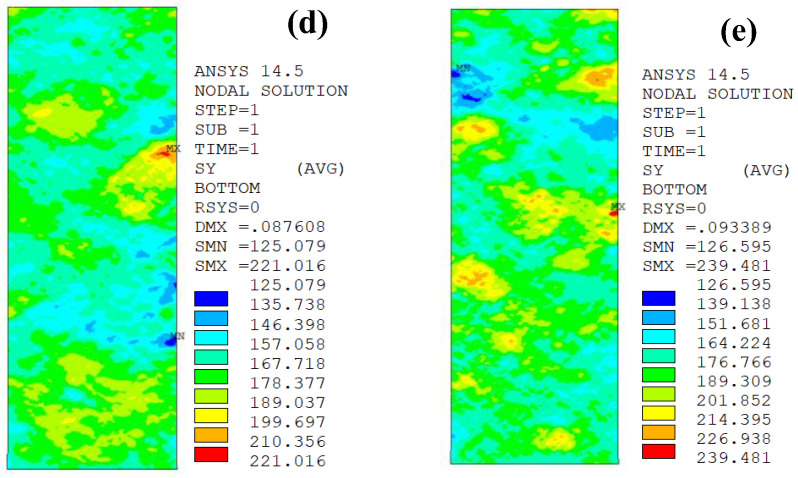
Peak tensile stress distribution on the unpatched corroded steel surfaces with various corrosion durations: (**a**) C0-1, (**b**) C6-1, (**c**) C9-1, (**d**) C15-1, (**e**) C18-1.

Figure 13a–e presents the peak tensile stress distribution on the unpatched corroded steel surfaces with various corrosion durations of 0, 6, 9, 15 and 18 months, respectively. As shown in Figure 13, the maximum tensile stress of the uncorroded steel plate appears at the junction of the parallel section and the transition section arc of the dog-bone specimen. Although the shape variation of the specimen leads to a certain extent of stress amplification, the stress concentration of the uncorroded steel plate can be almost ignored. The nominal tensile stress and the peak tensile stress are 140.332 MPa and 143.178 MPa, respectively, and the stress concentration factor is only 1.02. As for the unpatched corroded specimen, the degree of stress concentration caused by the rust pit has far exceeded the influence of the shape variation of the specimen. The peak tensile stress is located at the “critical pit” on the surface of the corroded steel plates, and the value of the peak stress increased with the corrosion duration increasing from 0 months to 6, 9, 15, and 18 months.

**Figure 14:**
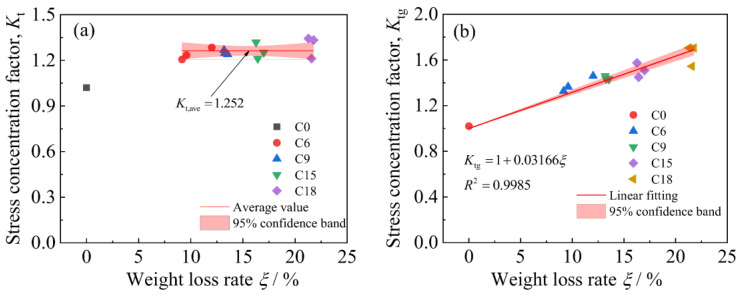
Effect of corrosion duration on the stress concentration of the unpatched corroded steel plate: (**a**) *K*_t_, (**b**) *K*_tg_.

Figure 14a,b illustrates the effect of the corrosion duration on the stress concentration factors *K*_t_ and *K*_tg_ of the unpatched corroded steel plate, respectively. As shown in Figure 14a, the stress concentration factor (*K*_t_) presents a significant increase with the corrosion duration increased from 0 month to 6 months. The corrosion duration continues to increase from 6 months to 18 months; however, it fluctuates up and down with no obvious tendency, and even the stress concentration factor (*K*_t_) of the corroded steel plate with the same corrosion duration shows a large dispersion. The value of *K*_t_ for the unpatched corroded steel plate with a corrosion duration of 6~18 months and a weight loss rate of 9.16~21.78% are approximately 1.205~1.343, the mean value is 1.252, and the upper and lower limit of the 95% confidence interval are 1.231 and 1.293, respectively. The convert stress concentration factor (*K*_tg_) of the unpatched corroded steel plate, as shown in Figure 14b, increases significantly with the increase in corrosion duration, and it presents a good linear relationship with the weight loss rate of the corroded steel plates:

(19) $$K_{\mathrm{tg}}=1+0.03166\xi$$

To interpret the above phenomenon, the stress concentration factor (*K*_t_), which reflects the intensity of the stress disturbance caused by the sudden change in geometric size near the rust pit, is conceptually the ratio of the peak tensile stress of the steel plate to the nominal stress of the net section of the corroded steel plate that corresponds to the position of the peak stress. Theoretically, *K*_t_ is only related to the shape, size, and position of the rust pits, but not to the degree of uniform corrosion of the corroded steel plate. Meanwhile, the effect of the corrosion duration on the 3D roughness parameters of the corroded steel surface, which is presented in Figure 5, indicates that local pitting corrosion and uniform corrosion alternately played the dominant role during the corrosion process, and, consequently, the stress concentration factor (*K*_t_) fluctuates up and down with the increase in corrosion duration. The concept of the converted stress concentration factor (*K*_tg_) is different from that of the stress concentration factor (*K*_t_); it reflects the ratio of the peak tensile stress of the corroded steel plate to the distal gross stress of the uncorroded steel plate. Since the external applied load is constant, the distal gross stress can be considered to be constant, whereas the peak tensile stress of the corroded steel plate is significantly affected by the corrosion duration. From this perspective, the converted stress concentration factor (*K*_tg_) has more practical significance in describing the influence of corrosion on the peak stress of the corroded steel plate.

### 5.2. Effect of CFRP Strengthening Stiffness

**Figure 15:**
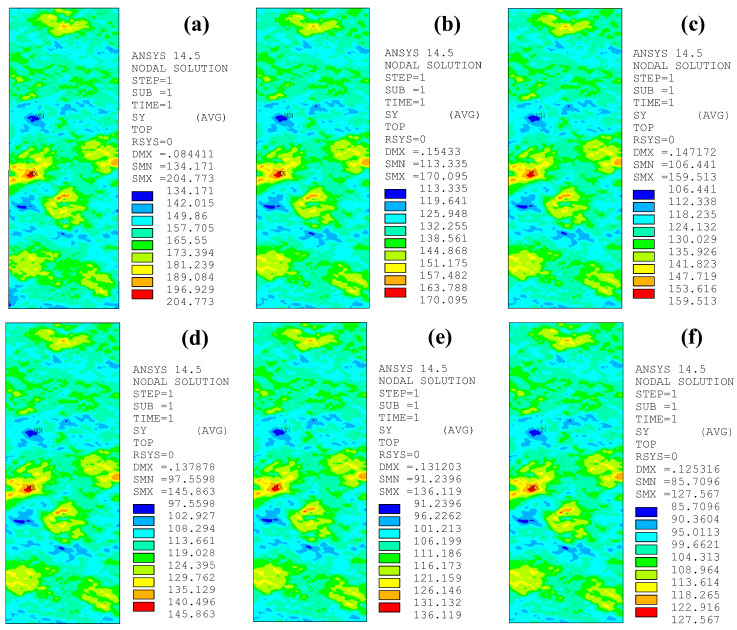
Peak tensile stress distribution on the corroded steel surfaces of the patched specimens with various CFRP strengthening stiffnesses: (**a**) C9-1, (**b**) C9-DS1.0-A1.0, (**c**) C9-DS1.4-A1.0, (**d**) C9-DS2.0-A1.0, (**e**) C9-DS2.5-A1.0, (**f**) C9-DS3.0-A1.0.

Figure 15 depicts the peak tensile stress distribution on the corroded steel surfaces of the patched specimens with various CFRP strengthening stiffnesses, where the corrosion duration of the corroded steel plate and the adhesive thickness of the models in Figure 15 are 9 months and 1.0 mm, respectively, and the thickness of the external patched CFRP plates increases from 0 mm to 1.0, 1.4, 2.0, 2.5, and 3.0 mm. As Figure 15 shows, the peak value of the tensile stress on the corroded steel surfaces decreases significantly, whereas the features of stress distribution and the stress concentration site do not change with the increase in strengthening stiffness ratio.

**Figure 16:**
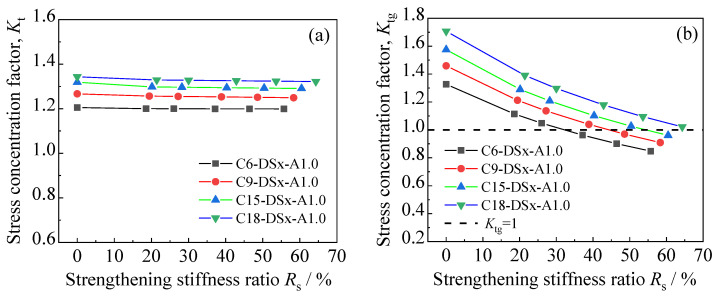
Influence of strengthening stiffness ratio on the stress concentration factors (**a**) *K*_t_ and (**b**) *K*_tg_ of the corroded steel plates strengthened with CFRP plates.

Figure 16a,b presents the influence of the strengthening stiffness ratio on the stress concentration factors *K*_t_ and *K*_tg_ of the corroded steel plates strengthened with CFRP plates, respectively. As presented in Figure 16a, the stress concentration factor (*K*_t_) of the patched corroded steel plates presents an extremely small decreasing trend with the increase in the strengthening stiffness of the CFRP plates. Taking the specimen with a corrosion duration of 15 months as an example, when the strengthening stiffness ratio increases from 0 (unpatched) to 60.46%, *K*_t_ decreases from 1.319 to 1.291, with a variation of only 2.13%, indicating that the variation in the stiffness of the external patched CFRP plates has little effect on the stress concentration factor (*K*_t_) of the corroded steel plate strengthened with CFRP plates. It can be seen in Figure 16b that the converted stress concentration factor (*K*_tg_) decreases continuously with the increase in the strengthening stiffness of the external patched CFRP plates, and the value of *K*_tg_ can even be reduced below 1.0 with an appropriate strengthening stiffness ratio. Conceptually, *K*_tg_ reflects the ratio of the peak tensile stress of the corroded steel plate strengthened with CFRP plates to the distal gross stress of the unpatched uncorroded steel plate, and when the value of *K*_tg_ decreases to 1.0, this means that the peak tensile stress of the corroded steel plate can be reduced below the stress level of the uncorroded steel plate under the same load condition through external patching CFRP plates to the substrates of the corroded steel plate. This indicates that by increasing the strengthening stiffness of the CFRP plates, the fatigue performance of corroded steel plate can be restored or can even exceed the state of the uncorroded steel plate.

### 5.3. Effect of Adhesive Thickness

**Figure 17:**
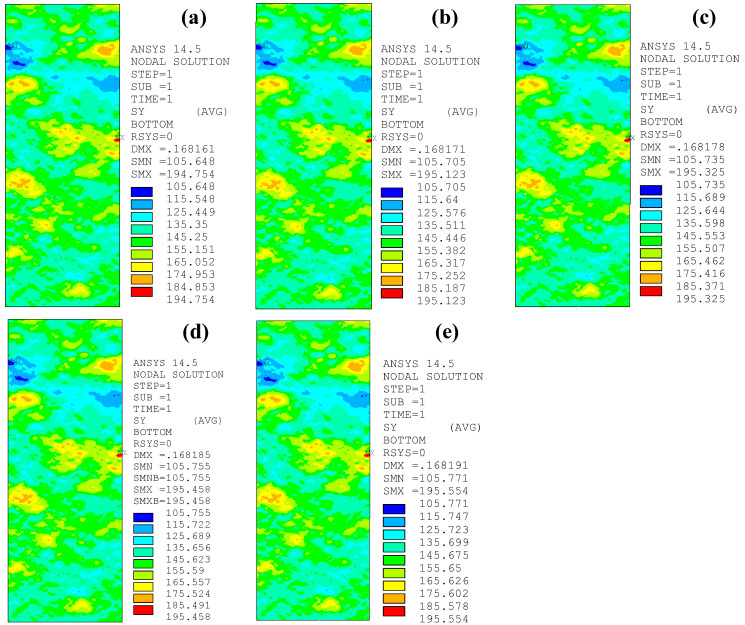
Peak tensile stress distribution on the corroded steel surfaces of the patched specimens with various adhesive thicknesses: (**a**) C18-DS1.0-A0.5, (**b**) C18-DS1.0-A1.0, (**c**) C18-DS1.0-A1.5, (**d**) C18-DS1.0-A2.0, (**e**) C18-DS1.0-A2.5.

**Figure 18:**
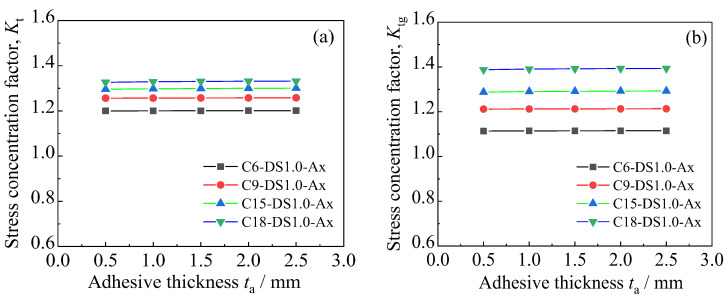
Effect of adhesive thickness on the stress concentration factors (**a**) *K*_t_ and (**b**) *K*_tg_ of the corroded steel plates strengthened with CFRP plates.

Figure 17 presents the peak tensile stress distribution on the corroded steel surfaces of the patched specimens with various adhesive thicknesses. Figure 18a,b shows the effect of the adhesive thickness on the stress concentration factors *K*_t_ and *K*_tg_ of the corroded steel plates strengthened with CFRP plates, respectively. Observations and interpretations based Figure 17 and Figure 18 may be summarized as follows: the peak tensile stress, and the stress concentration factors *K*_t_ and *K*_tg_ present a very small increasing trend with the increase in adhesive thickness. Taking the corroded steel plate specimen with a corrosion duration of 18 months as an example, when the thickness of the adhesive layer increases from 0.5 mm to 2.5 mm, the peak tensile stress of the corroded steel plates strengthened with CFRP plates increases from 194.754 MPa to 195.554 MPa, the corresponding stress concentration factor (*K*_t_) increases from 1.327 to 1.332, and the converted stress concentration factor (*K*_tg_) increases from 1.388 to 1.394. This indicates that although increasing the adhesive thickness is unfavorable for improving the strengthening effectiveness of the patched corroded steel plate, the adverse effect is very small. To interpret this phenomenon, the basis of the cooperative work of the corroded steel plate and CFRP plates is the load transfer of the adhesive layer, and the greater the thickness of the adhesive layer, the smaller the shear stiffness and the lower the load transfer efficiency of the interface between the corroded steel plate and the CFRP plates, and thus the worse the strengthening effectiveness. On the other hand, there is an effective bonding length on the bonding interface between the corroded steel plate and the CFRP plates [25,26]. Although the increase in adhesive thickness will lead to an increase in the effective bonding length of the CFRP plate, the strengthening effectiveness of the CFRP plates can still be fully exerted, since the bonding length of the CFRP plates in the models of this study is greater than the effective bonding length. The reduction in load transfer efficiency that is caused by the increase in adhesive thickness has only a certain impact on the area within a certain range on the end of the CFRP plates, and thus the influence of various adhesive thicknesses on the interfacial load transfer and strengthening effectiveness can be ignored.

### 5.4. Effect of Prestress Level of CFRP Plates

**Figure 19:**
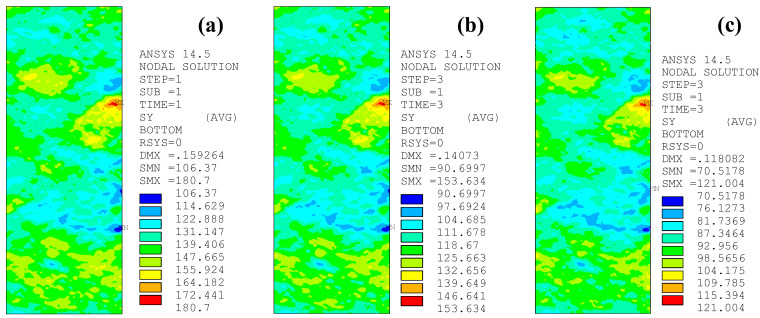

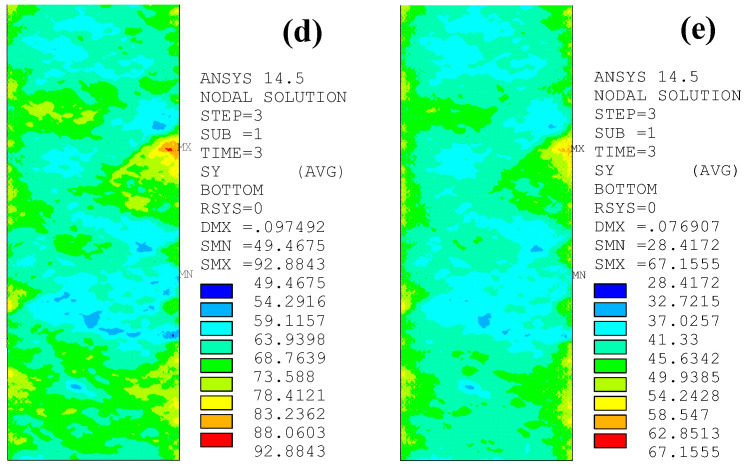
Peak tensile stress distribution on the corroded steel surfaces of the patched specimens with various prestress levels of CFRP plates: (**a**) C15-DS1.0-A0.5, (**b**) C15-DS1.0-A0.5-PS4.5, (**c**) C15-DS1.0-A0.5-PS10, (**d**) C15-DS1.0-A0.5-PS15, (**e**) C15-DS1.0-A0.5-PS20.

Figure 19 illustrates the peak tensile stress distribution on the corroded steel surfaces of the patched specimens with various prestress levels of CFRP plates, where the corrosion duration of the corroded steel plate, the strengthening stiffness ratio of external patched CFRP plates, and the adhesive thickness of the models in Figure 18 are 15 months, 0.5 mm, and 20.15%, respectively. The prestress level *α* is defined as the ratio of the prestress to the tensile strength of the CFRP plate varying from 0 to 4.5%, 10%, 15%, and 20%. The temperature reduction method with a temperature linear expansion coefficient of the CFRP plate of 1 × 10^−5^/°C is adopted to apply prestress to the CFRP plates, and the corresponding temperature load is 0, −65.45, −145.45, −218.18, and −290.91 °C, respectively. As Figure 19 illustrates, the peak tensile stress of the corroded steel plates strengthened with CFRP plates decreases continuously, whereas the feature of stress distribution and the stress concentration site on the surface of the corroded steel plate do not change with the increase in the prestress level of the external patched CFRP plates.

**Figure 20:**
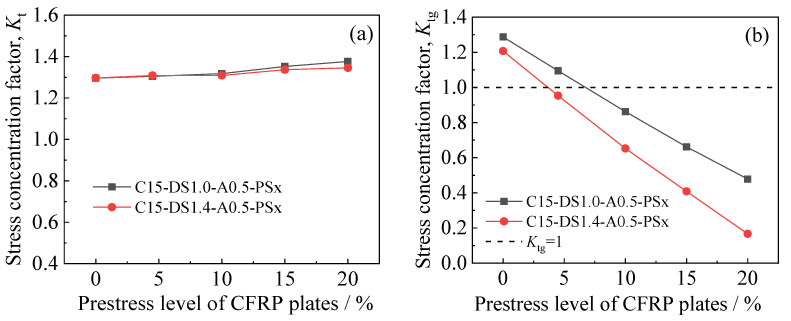
Effect of the prestress level of CFRP plates on the stress concentration factors (**a**) *K*_t_ and (**b**) *K*_tg_ of the corroded steel plates strengthened with CFRP plates.

Figure 20a,b presents the effect of the prestress level of the CFRP plates on the stress concentration factors *K*_t_ and *K*_tg_ of the corroded steel plates strengthened with CFRP plates, respectively. As shown in Figure 20a, with respect to the external patched specimens with the same corrosion duration and different strengthening stiffnesses and prestress levels of CFRP plates, the stress concentration factor (*K*_t_) is basically the same, indicating that *K*_t_ mainly depends on the surface morphology of the corroded steel plate and is hardly affected by the stiffness and prestress level of the external patched CFRP plates. It can be seen in Figure 20b that the converted stress concentration factor (*K*_tg_) presents a considerable decrease with the increase in the prestress level of the CFRP plates, and the greater the strengthening stiffness of the CFRP plates, the more significant the decrease amplitude of *K*_tg_. Taking the specimens with strengthening stiffnesses of 20.15% (i.e., C15-DS1.0-A0.5-PSx series specimens) and 28.21% (i.e., C15-DS1.4-A0.5-PSx series specimens) as an example, with the prestress level of CFRP plates increasing from 0 to 20%, the corresponding value of *K*_tg_ decreases from 1.288 and 1.208 to 0.479 and 0.167, with an amplitude of 62.81% and 86.18, respectively. The aforementioned phenomenon shows that the prestressing of CFRP plates, especially for specimens with a large strengthening stiffness ratio, has a significant impact on reducing the peak tensile stress of the corroded steel plate strengthened with CFRP plates. The larger the prestress level, the higher the utilization efficiency of the CFRP plates and the more significant the strengthening effectiveness of the corroded steel plate strengthened with CFRP plates.

## 6. Conclusions

In the present study, an accelerated corrosion experiment was first executed to acquire corroded steel plates, and an outdoors exposure test method for periodic water spray was adopted to simulate the corrosion process of steel structures in a general marine atmospheric environment. The surface profile measurements were conducted to obtain the 3D coordinate data of the corroded steel surface, and the topographic feature parameters of the corroded steel surface were calculated and analyzed. Finite element models considering the surface morphology of the corroded steel plate and the interfacial bonding properties between CFRP plates and corroded steel plates were established to investigate the stress concentration of the corroded steel plates strengthened with CFRP plates. The effects of corrosion duration, CFRP strengthening stiffness, adhesive thickness, and the prestress level of CFRP plates on the features of stress distribution and stress concentration factors *K*_t_ and *K*_tg_ of the corroded steel plate strengthened with CFRP plates were analyzed. Based on experimental and numerical analysis results, the following conclusions can be made within the scope of this study:

(1) The roughness of the corroded steel plates was characterized by the 3D roughness parameters *S*_a_, *S*_q_, and *S*_z_. Local pitting corrosion and uniform corrosion alternately played the dominant role in the surface topography and roughness of the steel substrates, resulting in the fluctuating variation process of *S*_a_, *S*_q_, and *S*_z_. Nevertheless, the values of *S*_a_, *S*_q_, and *S*_z_ of the corroded steel plates were much greater than that of the uncorroded ones.
(2) The features of stress distribution and stress concentration factor *K*_t_ of the corroded steel plate strengthened with and without CFRP plates are only related to the shape, size, and position of the rust pits on the surface of the corroded steel plate, but not to the degree of uniform corrosion of the corroded steel plate, nor the thickness of the adhesive layer, the strengthening stiffness and prestress level of the CFRP plates, or other reinforcement parameters. The value of *K*_t_ for the corroded steel plate with a corrosion duration of 6~18 months and a weight loss rate of 9.16~21.78% is approximately 1.199~1.345.
(3) The converted stress concentration factor *K*_tg_, which is defined as the ratio of the peak stress at the rust pits on the surface of the corroded steel plate to the gross stress of the unpatched, uncorroded steel plate, has more practical significance than the stress concentration factor *K*_t_ in describing the influence of corrosion (negative part) and CFRP plate reinforcement (positive part) on the peak tensile stress of the corroded steel plate strengthened with CFRP plates. The value of *K*_tg_ increases linearly with the increase in the weight loss rate of the corroded steel plate and decreases appreciably with the increase in the strengthening stiffness and prestress level of the CFRP plates, and it presents a very small increasing trend with the increase in adhesive thickness.

## Figures and Tables

**Table 1:** Results summary of surface profile measurements and stress concentrations analysis of the unpatched corroded steel plates.

Specimen No.	*C*_d_ /Month	*ξ*/ %	*S*_a_ /um	*S*_q_ /um	*S*_z_ /um	$\sigma_{\mathrm{peak}}$ /MPa	$\sigma_{\mathrm{nominal}}$ /MPa	*K*_t_	*K*_tg_
C0-1	0	0	15.87	20.37	206.36	143.178	140.332	1.020	1.020
C0-2	0	0	25.70	32.34	236.82	143.178	140.332	1.020	1.020
C0-3	0	0	18.19	23.58	167.67	143.178	140.332	1.020	1.020
C6-1	6	9.16	106.80	135.83	1130.96	186.192	154.483	1.205	1.327
C6-2	6	9.59	114.40	146.50	983.09	191.513	155.218	1.234	1.365
C6-3	6	12.02	102.48	133.12	1452.25	204.863	159.505	1.284	1.460
C9-1	9	13.19	133.44	169.29	1139.45	204.773	161.654	1.267	1.459
C9-2	9	13.25	177.37	217.42	1306.58	201.470	161.766	1.245	1.436
C9-3	9	13.54	139.36	176.69	1199.03	200.937	162.309	1.238	1.432
C15-1	15	16.26	131.52	168.46	1372.92	221.016	167.581	1.319	1.575
C15-2	15	16.42	136.70	165.30	1016.48	203.388	167.902	1.211	1.449
C15-3	15	16.99	141.74	173.59	1103.79	211.497	169.055	1.251	1.507
C18-1	18	21.28	133.00	174.71	1369.64	239.481	178.276	1.343	1.707
C18-2	18	21.58	133.02	166.40	1016.48	216.714	178.940	1.211	1.544
C18-3	18	21.78	162.38	211.22	1429.16	239.180	179.407	1.333	1.704

Notes: *C*_d_ is corrosion duration; *ξ* is weight loss rate that can be calculated by Equation (4); *S*_a_, *S*_q_ and *S*_z_ are the arithmetic mean height, the root mean square height, and the maximum height, respectively; $\sigma_{\mathrm{peak}}$ is the peak stress at the rust pit; $\sigma_{\mathrm{nominal}}$ is the nominal stress of the net section.

**Table 2:** Results summary of stress concentrations analysis of corroded steel plate strengthened with CFRP plates.

Specimen No.	*C*_d_ /Month	*ξ* /%	*R*_s_ /%	*t*_a_ /mm	*α*/%	$\sigma_{\mathrm{peak}}/\mathrm{MPa}$	$\sigma_{\mathrm{nominal}}$ /MPa	*K*_t_	*K*_tg_
C6-DS1.0-A0.5	6	9.16	18.58	0.5	-	156.300	130.280	1.200	1.114
C6-DS1.0-A1.0	6	9.16	18.58	1.0	-	156.376	130.280	1.200	1.114
C6-DS1.0-A1.5	6	9.16	18.58	1.5	-	156.415	130.280	1.201	1.115
C6-DS1.0-A2.0	6	9.16	18.58	2.0	-	156.441	130.280	1.201	1.115
C6-DS1.0-A2.5	6	9.16	18.58	2.5	-	156.462	130.280	1.201	1.115
C6-DS1.4-A1.0	6	9.16	26.01	1.0	-	147.106	122.597	1.200	1.048
C6-DS2.0-A1.0	6	9.16	37.16	1.0	-	135.106	112.633	1.200	0.963
C6-DS2.5-A1.0	6	9.16	46.44	1.0	-	126.523	105.489	1.199	0.902
C6-DS3.0-A1.0	6	9.16	55.73	1.0	-	118.961	99.197	1.199	0.848
C9-DS1.0-A0.5	9	13.19	19.44	0.5	-	170.025	135.343	1.256	1.212
C9-DS1.0-A1.0	9	13.19	19.44	1.0	-	170.095	135.343	1.257	1.212
C9-DS1.0-A1.5	9	13.19	19.44	1.5	-	170.146	135.343	1.257	1.212
C9-DS1.0-A2.0	9	13.19	19.44	2.0	-	170.186	135.343	1.257	1.213
C9-DS1.0-A2.5	9	13.19	19.44	2.5	-	170.219	135.343	1.258	1.213
C9-DS1.4-A1.0	9	13.19	27.22	1.0	-	159.513	127.070	1.255	1.137
C9-DS2.0-A1.0	9	13.19	38.88	1.0	-	145.863	116.398	1.253	1.039
C9-DS2.5-A1.0	9	13.19	48.60	1.0	-	136.119	108.784	1.251	0.970
C9-DS3.0-A1.0	9	13.19	58.32	1.0	-	127.567	102.106	1.249	0.909
C15-DS1.0-A0.5	15	16.26	20.15	0.5	-	180.700	139.473	1.296	1.288
C15-DS1.0-A0.5-PS4.5	15	16.26	20.15	0.5	4.5	153.634	117.767	1.305	1.095
C15-DS1.0-A0.5-PS10	15	16.26	20.15	0.5	10	121.004	91.839	1.318	0.862
C15-DS1.0-A0.5-PS15	15	16.26	20.15	0.5	15	92.884	68.690	1.352	0.662
C15-DS1.0-A0.5-PS20	15	16.26	20.15	0.5	20	67.156	48.800	1.376	0.479
C15-DS1.0-A1.0	15	16.26	20.15	1.0	-	181.023	139.473	1.298	1.290
C15-DS1.0-A1.5	15	16.26	20.15	1.5	-	181.193	139.473	1.299	1.291
C15-DS1.0-A2.0	15	16.26	20.15	2.0	-	181.305	139.473	1.300	1.292
C15-DS1.0-A2.5	15	16.26	20.15	2.5	-	181.387	139.473	1.301	1.293
C15-DS1.4-A0.5-PS4.5	15	16.26	28.21	0.5	4.5	133.786	102.271	1.308	0.953
C15-DS1.4-A0.5-PS10	15	16.26	28.21	0.5	10	91.627	70.023	1.309	0.653
C15-DS1.4-A0.5-PS15	15	16.26	28.21	0.5	15	57.313	42.886	1.336	0.408
C15-DS1.4-A0.5-PS20	15	16.26	28.21	0.5	20	23.377	17.377	1.345	0.167
C15-DS1.4-A1.0	15	16.26	28.21	1.0	-	169.456	130.704	1.296	1.208
C15-DS2.0-A1.0	15	16.26	40.31	1.0	-	154.607	119.440	1.294	1.102
C15-DS2.5-A1.0	15	16.26	50.38	1.0	-	144.051	111.436	1.293	1.026
C15-DS3.0-A1.0	15	16.26	60.46	1.0	-	134.811	104.438	1.291	0.961
C18-DS1.0-A0.5	18	21.28	21.44	0.5	-	194.754	146.803	1.327	1.388
C18-DS1.0-A1.0	18	21.28	21.44	1.0	-	195.123	146.803	1.329	1.390
C18-DS1.0-A1.5	18	21.28	21.44	1.5	-	195.325	146.803	1.331	1.392
C18-DS1.0-A2.0	18	21.28	21.44	2.0	-	195.458	146.803	1.331	1.393
C18-DS1.0-A2.5	18	21.28	21.44	2.5	-	195.554	146.803	1.332	1.394
C18-DS1.4-A1.0	18	21.28	30.01	1.0	-	182.043	137.120	1.328	1.297
C18-DS2.0-A1.0	18	21.28	42.88	1.0	-	165.395	124.775	1.326	1.179
C18-DS2.5-A1.0	18	21.28	53.60	1.0	-	153.655	116.067	1.324	1.095
C18-DS3.0-A1.0	18	21.28	64.32	1.0	-	143.446	108.495	1.322	1.022

Notes: *C*_d_ is corrosion duration; *ξ* is weight loss rate; *R*_s_ is strengthening stiffness ratio; *α* is prestress level of CFRP plate; *t*_a_ is adhesive thickness; $\sigma_{\mathrm{peak}}$ is the peak stress at the rust pit; $\sigma_{\mathrm{nominal}}$ is the nominal stress of the net section.

## Data Availability

All data are shown in the paper.
